# Thermal evolution of the crystal structure and phase transitions of KNbO_3_

**DOI:** 10.1098/rsos.180368

**Published:** 2018-06-20

**Authors:** S. L. Skjærvø, K. Høydalsvik, A. B. Blichfeld, M.-A. Einarsrud, T. Grande

**Affiliations:** Department of Materials Science and Engineering, NTNU Norwegian University of Science and Technology, 7491 Trondheim, Norway

**Keywords:** thermal evolution, phase transitions, KNbO_3_, high-temperature powder X-ray diffraction

## Abstract

The thermal evolution of the crystal structure and phase transitions of KNbO_3_ were investigated by high-temperature powder X-ray diffraction and Rietveld refinement of the diffraction data. Two phase transitions from orthorhombic (*Amm*2) to tetragonal (*P*4*mm*) and from tetragonal to cubic ($Pm\bar{3}m$) were confirmed, both on heating and cooling. Both phase transitions are first order based on the observed hysteresis. The mixed displacive and order–disorder nature of the tetragonal to cubic transition is argued based on symmetry and apparent divergence of the atomic positions from pseudo-cubic values. The transition between the orthorhombic and tetragonal phase shows no temperature-dependence for atomic positions and only thermal expansion of the unit cell parameters and is thus discussed in relation to a lattice dynamical instability.

## 1. Introduction

The discovery of the ferroelectric properties of BaTiO_3_ in 1946 [1] led to an extended search for materials with similar properties and in 1951, Matthias & Remeika [2] showed the ferroelectric nature of potassium niobate, KNbO_3_. They found evidence for two temperature-induced phase transitions, from orthorhombic to tetragonal symmetry at 224°C, and from tetragonal to cubic at 434°C by dielectric spectroscopy. A few years later, Shirane *et al*. [3] confirmed these findings by measuring the specific heat anomalies at the transition temperatures in addition to finding a third transition temperature at −10°C, where the structure turns rhombohedral upon cooling. Following the discovery of lead zirconate titanate in 1954 [4], the interest for KNbO_3_ dropped, but was reignited in the early 2000s due to legislation introduced to reduce the use of lead in technological applications [5,6].

 In recent years the phase transitions of KNbO_3_ have been investigated by several different techniques including infrared spectroscopy [7–9], Raman spectroscopy [8–11], thermal expansion measurements [11,12], second harmonic generation [13] and luminescence measurements [14]. However, a complete set of unit cell parameters and insight into the crystal structure across the phase transitions have so far not been reported. Here, we present a detailed study of the thermal evolution of the crystal structure and temperature-induced phase transitions of KNbO_3_, upon heating and cooling, measured by *in situ* high-temperature powder X-ray diffraction.

## 2. Material and methods

Powder of KNbO_3_ was synthesized hydrothermally, following the recipe of Wang *et al.* [15]. A mixture of 0.5 g Nb_2_O_5_ (Sigma Aldrich, 99.99% trace metal basis), 18 g KOH (VWR, Emsure® Analytical reagent, ≥85%) and 20 g distilled H_2_O was heated for 6 h at 180°C in a 125 ml Teflon-lined stainless steel autoclave (4748 Large Capacity Autoclave, Parr Instrument Company, IL, USA). After thorough washing with ethanol and water, the powder was dried at 80°C overnight, and then the powder was thermally annealed at 1000°C for 5 h in a Nabertherm P330 furnace. The sintered powder was mortared for 15 min in an agate mortar.

An Al_2_O_3_ sample carrier was used to mount the KNbO_3_ powder sample into an in-house Bruker AXS D8 Advance diffractometer (Cu-k*α* source) and heated with a radiant heater. The rate of heating and cooling was 0.02°C s^−1^. The temperature was calibrated by refining the thermal expansion of an Al_2_O_3_ sample. The 2*θ* range of 19–60° was scanned with a step size of 0.016° with 1.25 s per step, giving a total collection time of 1 h per scan. Each scan was delayed by 10 s to ensure that the sample had reached the set temperature.

Rietveld refinements of the diffraction data were performed using Bruker AXS Topas 5 in launch mode and JEdit (v. 4.3.1) with macros for Topas [16]. The space group and structure model for the three polymorphs of KNbO_3_ used in the refinements are given in table 1. The displacement of the atomic positions of K and O for the two non-cubic structures was refined using the Δ-variables defined in table 1, while Nb was fixed to the pseudo-cubic position. The background was described with Chebychev polynomials of sixth order and the peak profiles were refined with fundamental parameters and Lorentzian strain broadening. The sample displacement and unit cell parameters were refined. Isotropic temperature factors were fixed to room temperature values determined by neutron diffraction [17]. The data obtained by the refinement are listed in separate tables (see Data accessibility).

**Table 1.** Atomic positions for the three space groups.

space group	atom	Wyckoff site	*x*	*y*	*z*
*Amm*2	K	2a	0	0	0 + ΔK_z_
	Nb	2b	½	0	½
	O(1)	2a	0	0	½ + ΔO1_z_
	O(2)	4e	½	¼ + ΔO2_y_	¼ + ΔO2_z_
*P*4*mm*	K	1a	0	0	0 + ΔK_z_
	Nb	1b	½	½	½
	O(1)	1b	½	½	ΔO1_z_
	O(2)	2c	½	0	½ + ΔO2_z_
$Pm\bar{3}m$	K	1a	0	0	0
	Nb	1b	½	½	½
	O	3c	0	½	½

## 3. Results and discussion

**Figure 1.**
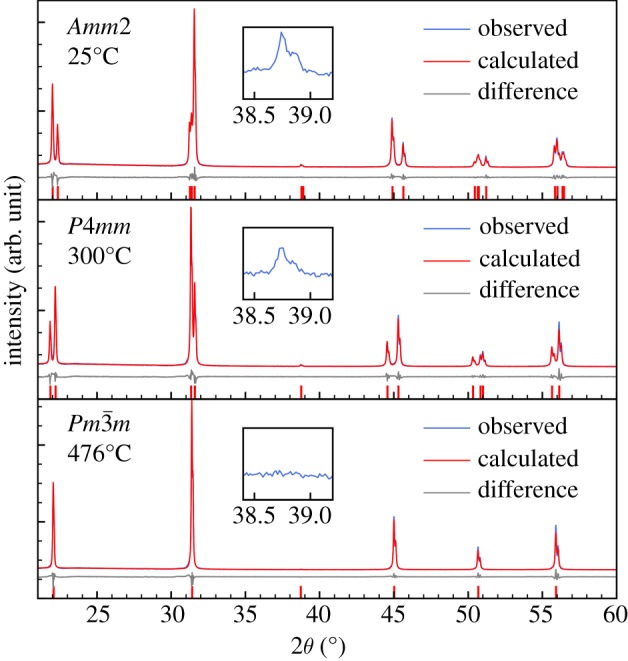
Measured and calculated diffraction patterns and the difference between these two at three different temperatures upon heating, representing each of the three polymorphs of KNbO_3_.

Representative X-ray diffraction patterns of KNbO_3_ as a function of temperature are presented in figure 1. The Bragg reflections shift to lower angles upon heating due to thermal expansion. The difference between the calculated diffraction pattern from the models obtained by Rietveld refinement and the three experimental diffraction patterns are also shown in figure 1. The Rietveld refinements are summarized in separate tables (see Data accessibility).

**Figure 2.**
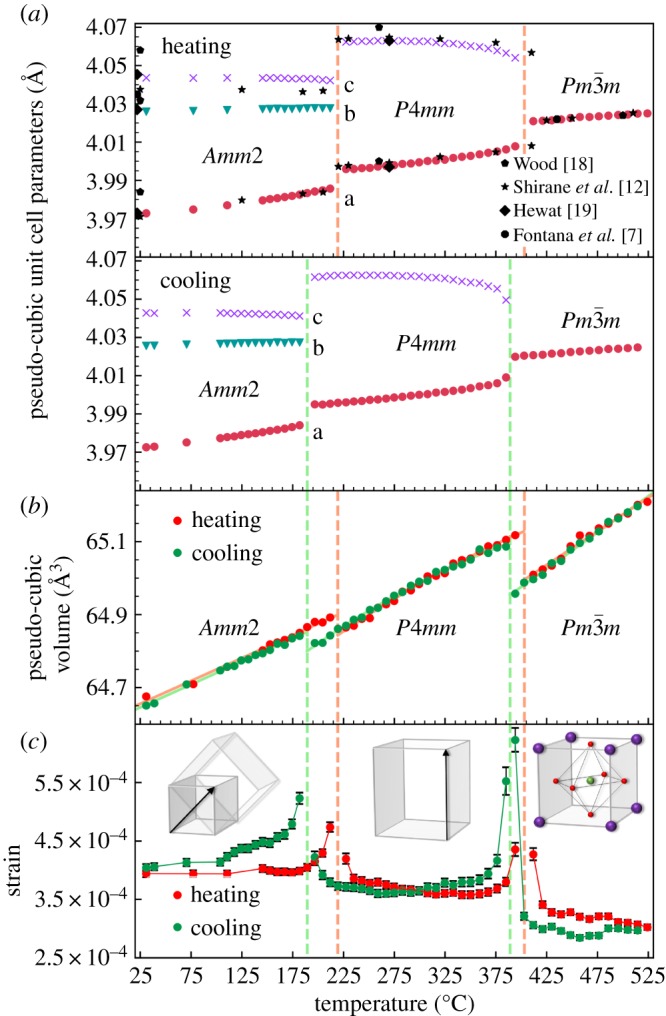
Pseudo-cubic (*a*) unit cell parameters and (*b*) unit cell volume of KNbO_3_ upon heating and cooling measured *in situ* with X-ray diffraction. Previous published data by Wood [18], Shirane *et al.* [12], Hewat [19] and Fontana *et al.* [7] are shown for comparison. (*c*) The refined strain.

For comparing the unit cell parameters across the three different space groups, the orthorhombic and tetragonal cells were transformed to a pseudo-cubic unit cell maintaining the individual axis but with the same orientation as the true cubic cell. These pseudo-cubic unit cell parameters, determined by Rietveld refinement, upon heating and cooling are presented in figure 2*a*. The unit cell parameters are in good accordance with previously reported data in the literature [7,12,18,19], but the present dataset has significantly higher accuracy, especially in terms of temperature resolution and the distortion of the unit cell. The two phase transitions are evident as reflections merge (split) due to increase (decrease) in symmetry (figure 1). The phase transitions occurred at 219 ± 7 and 403 ± 7°C upon heating and 189 ± 7 and 389 ± 7°C upon cooling, respectively. The unit cell volume, as shown in figure 2*b*, is discontinuous over both phase transitions, pointing to the transitions being first order. The unit cell volumes coincide upon heating and cooling, except around the phase transition temperatures where a hysteresis is observed. The observed hysteresis gives additional support for the first-order nature of the transitions. The hysteresis is especially large for the orthorhombic to tetragonal transition. The refined strain, presented in figure 2*c*, generally decreases upon heating, except around the phase transition temperatures where it diverges in line with expectations due to fluctuations near the transitions.

**Figure 3.**
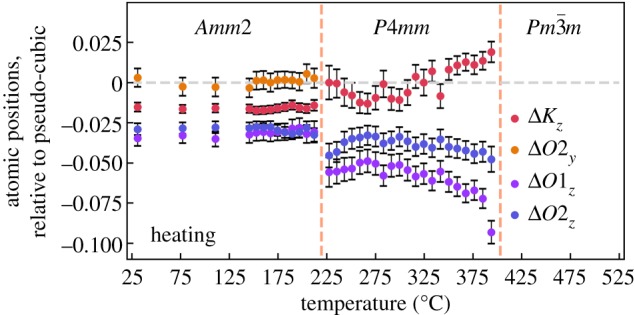
Deviation from pseudo-cubic atomic positions in the *Amm*2 and *P*4*mm* phases upon heating.

The transition between the tetragonal and cubic phase shows signs of being displacive as the tetragonal unit cell parameters slowly converge towards cubic when approaching the phase transition temperature. Assuming a displacive transition, one would also expect the atomic positions, presented in figure 3, to converge towards pseudo-cubic values upon heating. Instead, they diverge, inferring the signature of a significant order–disorder component, in addition to being displacive. This is analogous to literature on BaTiO_3_ [21,22], where an increasing ordering of the Ti atom towards the faces of the Ti–O octahedra upon cooling can be observed. Thus, the average crystal structure described by the Rietveld refinements cannot describe the local structure in KNbO_3_, explained in further detail for BaTiO_3_ by Egami & Billinge [23].

The transition between the orthorhombic and tetragonal phase cannot be displacive as there is no group–subgroup relationship between the two phases. This is supported by the unit cell parameters only being affected by the thermal expansion of the unit cell when approaching the transition temperature. Also, the deviation from pseudo-cubic of the atomic positions for the orthorhombic and tetragonal phases upon heating shows no significant temperature-dependence when approaching the transition between them. This is in agreement with the explanation of Fontana *et al.* [20], where the phase transition is argued from a lattice dynamical instability where the crystal is deformed and the direction of the spontaneous polarization changes.

## 4. Conclusion

The high-temperature X-ray diffraction study of KNbO_3_ confirmed the two phase transitions from orthorhombic (*Amm*2) to tetragonal (*P*4*mm*) and from tetragonal to cubic ($Pm\bar{3}m$) symmetry at 219 ± 7 and 403 ± 7°C upon heating and 189 ± 7 and 389 ± 7°C upon cooling, respectively. The converging unit cell parameters towards cubic values upon heating of the tetragonal phase point to a displacive nature of the transition from tetragonal to cubic, but based on the divergence of atomic positions we suggested a significant order–disorder component to the phase transition. The linear expansion of the orthorhombic unit cell and the lack of temperature-dependence on the atomic positions are in line with the absent group–subgroup relationship of the two polymorphs, and might be explained by a lattice dynamical instability.

## Data accessibility

The X-ray diffraction data, temperature calibration and parameters obtained by the Rietveld refinements are available at https://dx.doi.org/10.5061/dryad.td26kr2 [24].
